# Supplementary material for: Chromothripsis during telomere crisis is independent of NHEJ, and consistent with a replicative origin
Source: Genome Res. 2019 May;29(5):737–49. doi: 10.1101/gr.240705.118 (PMC6499312; doi:10.1101/gr.240705.118)
Supplement: Supplemental Material [file supp_gr.240705.118_Supplemental_file_1.zip › contigs/annotated_contigs/DB45/contig.3.DB45_length_1172_mean_cov_41.2482935154.docx]

**DB45_length_1172_mean_cov_41.2482935154**

```
AAGAAGAGAATGCGCATTTGGCTCTTCTTTAACACCAAGTCCACAAAAAAAATATTGGGTTTCCCC|TTTTTTTTTTTTTTTTTTTTCC
                                                                   >chr8:116421134-11642
CAACATATGAACTGCAGCATATCACTTTTTCTTTTTGTGCCTCAGGTTCCTCAGCTGTAAAATTGAAAAATATATGTATTAATAATATT
1509 - E=2e-204
ATTAATAATAATAATGGTAATGTAATACTTGTTTGTAAAGCACTTTGAGATCCTTGGTTGAAAGGCACCATAGGAGTGCCAAGTATTAT

TATGTGGCCAAGGGGGTTATTTAAACTGTCAGTTCCCAAAGGCCAGGAAAGGTTGGGGTCATTTTTCTTAAAGACGAGCTGTAAATATC

AACTAGGCAGCCAATAGTGTTGACTATGAAGATGCAAAACTATTACTAGGCTGATAAAATCATAGTTTCTTAATGGCTACCAATAA|AC

ACAATTTTTATTGTTAATCTTTCTGA|AGAAACTAGTATATATCTTTTTACACAAAATCTATCAGTAAGAGATTGATTGGTTAAAGAAA
                           >chr8:116420432-116420625 + E=6e-65 p=4e-02
AAAAGCAAAGCAAAACAAGTTGAGAGAATTCAGGCTTTATCAATATAAGTAATAATTTTTTAGAATGGTGATTTGATTTCACCATTTCA

ATTCAGCAGAGCCTGTATATATATATATATATGTATATA|TAT|GTTACGATGTGTAAAAACTCCTGCTTATGAAGTTTAACTTTGAAT
                                        >chr8:116419863-116420348 - E=6e-243
TCGGTATACTCATGTGAACATGATGCAATTCCTAATCAATCATCTCAAAAAGTTTAAATTAATATATCAAATATCTTATTTGTTTGGTT

AAAATCAAATTAATCAGGGCAAGTTCCCTGAAAGATGACTTTGAAAGAGAAATGAGGCAGAACAGATCCAGATAGGAAAGGACGTTGGC

AAGACTCACCTGCATGTTTTTAACCAGAAGGAAAAATACAAAAAGACAGAACATAAAGTATATAAATAATTGATTGCTTCAGAAGATTT

GCTTGGAGAGTTTAAAATAATTAGTATCTCCCCTCATTATACAATCTTCATTTCTCATTTTTGGGAGCCCAAATTAAGGAACAAATCCT

TCTAATTATTTTTTTTTTTTTTTTTTTTTTTTTTTTTTTTTTTTTTTTGAAATTAGCAGAGAGTCTTTGAGGGGCTTTCAT|TTTGGTT

ATGCCCTTTCTTTTCATCTGG
```
